# Identifying Key Indicators and Components of Earthquake Preparedness Exercises in the Prehospital Setting: An Exploratory Content Analysis From Paramedic Nurses’ Perspectives

**DOI:** 10.1155/nrp/1785701

**Published:** 2026-01-29

**Authors:** Asiye Aminafshar, Ali Khosravizad, Ali Sahebi, Mahmood Nekoeimoghadam, Mohammadreza Amiresmaili, Asghar Tavan, Hojjat Farahmandnia

**Affiliations:** ^1^ Modeling in Health Research Center, Institute for Futures Studies in Health, Kerman University of Medical Sciences, Kerman, Iran, kmu.ac.ir; ^2^ Non-Communicable Diseases Research Center, Ilam University of Medical Sciences, Ilam, Iran, medilam.ac.ir; ^3^ Health in Disasters and Emergencies Research Center, Institute for Futures Studies in Health, Kerman University of Medical Sciences, Kerman, Iran, kmu.ac.ir; ^4^ Student Research Committee, Kerman University of Medical Sciences, Kerman, Iran, kmu.ac.ir

**Keywords:** earthquake, exercise, nurse, paramedic, prehospital, preparedness

## Abstract

**Introduction:**

Design, implementation, and evaluation of prehospital exercises are some of the important steps to prepare and deploy natural disaster risk management programs, particularly concerning earthquake hazards. This study aimed to identify and provide valid operational indicators and components that can be utilized in the management and assessment of prehospital preparedness exercises in earthquakes.

**Method:**

A qualitative conventional content analysis constituted the methodological approach for this study. Data were systematically gathered through in‐depth, semistructured interviews with 11 paramedic nurses that were purposefully selected based on their demonstrated expertise in prehospital exercise management. Data analysis was done in five steps based on Granheim and Lundman’s approach, and for the trustworthiness of the data, this study used Lincoln and Guba’s recommendations.

**Result:**

After multiple rounds of analyzing and summarizing the data and taking into consideration similarities and differences, 375 initial codes, 12 subcategories, and 3 main categories were created based on the results of data analysis. The main categories included (A) “Strategic Organization of the Exercise”; (B) “Empowerment and Self‐Efficacy Enhancement of Exercise Personnel”; and (C) “Improvement and Development of Effective Exercise Drivers.”

**Conclusion:**

This research establishes a crucial knowledge framework that empowers exercise planners in prehospital settings to design scientifically sound and standardized exercises aimed at enhancing disaster response processes. The primary finding is that the successful implementation and evaluation of both discussion‐based and operation‐based exercises, when informed by these identified quality indicators, significantly fosters knowledge development and promotes essential behavioral change among prehospital paramedic nurses, thereby facilitating a standardized emergency response.

## 1. Introduction

Earthquakes are among the most unpredictable and catastrophic natural disasters capable of causing mass casualties, severe destruction of urban and rural infrastructure, and high mortality with no advanced notice [1]. Prehospital emergency services, which involve providing immediate help and moving patients, are essential during earthquake rescues, and quick medical help can greatly increase the chances of survival for those injured [2]. However, these services often face major challenges due to the destruction caused by earthquakes to buildings and health systems [3]. The absence of prehospital emergency services after an earthquake quickly closes the survival window for severely injured patients and leads to an increase in preventable mortality. This situation simultaneously causes immediate saturation of hospital capacity and disruption of vital care chains [4]. Prehospital paramedic personnel face a range of challenges during disasters that can affect their professional performance, subsequently influencing the efficiency and quality of care provided [5].

Multiple studies identify key challenges facing prehospital emergency teams in the response process to natural disasters [6, 7]. Bijani et al. and Kuday et al. highlight the absence of a disaster management plan, lack of standardized disaster medical assistance teams, and overall resource shortages, while the presence of skilled medical volunteers and military medical teams can improve service delivery [5, 8]. Alyami et al. declared key difficulties in managing emergency medical services at the scene of a mass casualty incident are designing and implementing a functional medical response strategy outside the hospital, providing necessary care despite severe shortages in personnel and supplies, and creating a long‐term strategy for ongoing field medical support [9].

The medical management of earthquakes follows the same principles as other disasters, whether natural or man‐made, and encompasses the four stages of preparedness, mitigation, response, and recovery [10, 11].

Preparedness for earthquakes involves constructing well‐designed buildings that can resist seismic shaking and fostering a resilient community capable of coping with disasters and returning to normal function as quickly as possible [12]. One way to reduce damage caused by earthquakes is to improve the preparedness of prehospital systems for a correct and timely response. On the other hand, the most important way to create, maintain, and increase preparedness is to design, perform, and evaluate disaster exercises [13]. Studies conducted on assessing the functional preparedness of hospitals and prehospital during disasters through exercise suggest that the selection of key indicators for disaster exercises should be considered during the design phase of exercise [14, 15]. Additionally, the selection of indicators should be based on the expected effectiveness and efficiency of each different section separately. Furthermore, in the development, conduct, and evaluation of disaster exercises, the satisfaction indicators for each expected performance should be determined [16].

Qualitative research serves as a critical tool for gaining an in‐depth understanding of complex phenomena. It provides a methodology to explore the nuanced perspectives of prehospital emergency specialists, enabling the identification of key components and the development of indicators tailored to the nation’s specific cultural, organizational, and social context [17].

So far, no study has extracted indicators of the design, implementation, and evaluation of prehospital exercise for earthquake hazards from the perspective of operational personnel. Therefore, this study aimed to identify and provide valid operational indicators and components that can be utilized in the management and assessment of prehospital response exercises in earthquake hazards. The indicators and components highlighted in this study are anticipated to enhance the effectiveness of prehospital exercises, improve team coordination, reduce performance errors, and elevate the quality of prehospital emergency care during emergencies and disasters.

## 2. Method

### 2.1. Study Design and Setting, and Participants

This qualitative study employed a conventional content analysis approach to identify the essential components for the design, implementation, and evaluation of prehospital earthquake preparedness exercises in 2025. This study was conducted through semistructured interviews with key informants, using purposive sampling with 11 paramedic nurses who had rich experience in the field of management disaster exercises in prehospital systems.

### 2.2. Inclusion and Exclusion Criteria of the Study

The inclusion criteria consisted of substantial experience in designing, implementing, and evaluating prehospital preparedness exercises in disaster contexts. Individuals who were unwilling to participate or unable to continue participation throughout the study were excluded. This qualitative study began with broad questions. Members of the research team participated in developing the interview guide. Two pilot interviews were conducted with individuals outside the primary sample to assess conceptual validity and ensure the clarity and interpretability of the questions from participants’ perspectives. Feedback from these pilots, together with the research team’s notes, informed the final revisions to the interview guide. Depending on the interview trajectory, probing and guiding questions were employed to elicit deeper insights into participants’ experiences and viewpoints. Interviews were conducted between March 5 and June 7, 2025, at locations chosen by participants where they felt comfortable. The main questions were encompassed:

• What are the essential components and characteristics of operations‐based and discussion‐based exercises?
• What requirements and functions should be incorporated into the design, implementation, and immediate postexercise phases?
• What obstacles and challenges have you faced in implementing a discussion‐based and operations‐based exercise with global standards?

Exploratory questions, like “Can you give an example of a problem or difficulty you encountered?” or “Can you explain this topic in more detail?” were used to understand the issues and ideas being talked about more clearly. The interviews lasted around 60–90 min, with an average time of 75 min. In this study data collection continued until saturation was achieved. Although there is no universally agreed‐upon definition of data saturation and no standardized method for applying it, it is commonly understood as an iterative process of adding new participants to the dataset until additional data no longer yield new information [18]. Data saturation was reached by the ninth interview; however, two additional confirmatory interviews were conducted to ensure data adequacy and verify that no new codes were identified.

### 2.3. Ethical Considerations

Ethical considerations in this study encompassed communicating the study’s significance, goals, and methods; offering voluntary participation; audio recording interviews; protecting data confidentiality at all stages; obtaining written consent; and mutually agreeing on the interview’s scheduling and location. Participants were assured that the information collected would be used exclusively for research purposes. Throughout the study, researchers worked to anonymize data, safeguard participants’ identities, and keep personal information secure.

### 2.4. Data Analysis

**Figure 1:**
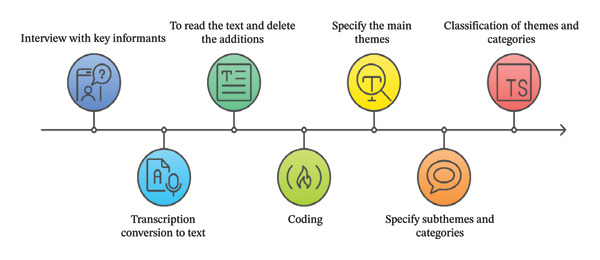
The steps of qualitative data analysis.

Data analysis was conducted following the five‐step approach proposed by Graneheim and Lundman [19]. First, the recorded interviews were transcribed verbatim. To ensure accuracy, each transcript was meticulously compared with the corresponding audio file. In the second step, the entire text of each interview was read multiple times to achieve a holistic understanding of its content. Third, meaning units relevant to the research questions were identified, condensed, and systematically coded. In the fourth step, codes with similar meanings were grouped and organized into subcategories based on their shared properties. Finally, through a process of constant comparison and theoretical abstraction, the main categories were formulated by integrating and refining the subcategories. The qualitative data analysis was facilitated using MAXQDA software (Version 20). The steps of qualitative data analysis are specified in Figure 1.

### 2.5. Reliability and Validity

To ensure the rigor and validity of the findings, the trustworthiness criteria for qualitative research established by Guba and Lincoln: credibility, dependability, confirmability, and transferability were employed [20]. To enhance credibility, strategies such as prolonged engagement in the field and persistent observation were utilized. Furthermore, the extracted codes and categories were reviewed and verified by the research team through peer debriefing sessions. The conceptual validity of the findings was also confirmed by seeking feedback from some participants on the developed categories and interpretations (member checking). To promote confirmability, an audit trail was maintained, ensuring that the research process and findings were traceable and that researcher bias was minimized. All phases of data collection, analysis, and interpretation were systematically reviewed and approved by the entire research team.

## 3. Results

### 3.1. Demographic Information of Participants

The study included 11 participants, all of whom had direct experience in conducting prehospital exercises. The majority of participants were male (*n* = 8, 83%) and held a doctoral degree (*n* = 9, 82%). Senior Iranian Emergency Medical Services (EMS) officers with doctoral degrees were selected based on their expertise in designing, conducting, and evaluating prehospital disaster exercises. All 11 participants (100%) reported previous experience in responding to various types of incidents and disasters and had been involved in prehospital drills. The demographic characteristics of the participants are summarized in Table 1.

**Table 1:** Demographic information of participants.

Demographic characteristics	Subcategory	Number (%)
Gender	Male	8 (83)
	Female	3 (17)

Age (Years)	40–50	5 (45.5)
	≥ 50	6 (54.5)

Work experience (Years)	11–20	3 (17)
	21–30	8 (83)

Organizational positions	Operational paramedic nurse	2 (18)
	Administrative paramedic nurse	9 (82)

Educational status	Doctoral degrees	9 (82)
	Master’s	2 (18)

### 3.2. Main Results

After multiple rounds of analyzing and summarizing the data and taking into consideration similarities and differences, 375 initial codes, 12 subcategories, and 3 main categories were created based on the results of data analysis. The main categories included (A) “Strategic Organization of the Exercise”; (B) “Empowerment and Self‐Efficacy Enhancement of Exercise Personnel”; and (C) “Improvement and Development of Effective Exercise Drivers.” The details of the main categories and subcategories are presented in Table 2.

**Table 2:** Main categories and corresponding subcategories.

Main category	Subcategory	Example of codes	Meaning unit
Strategic organization of the exercise	Exercise command chain	‐ Definition of roles and responsibilities in disaster management ‐ Adherence to the chain of command ‐ Strengthening incident command at the scene	‐ Unified command during crises and avoidance of parallel or duplicated actions ‐ Clearly defined positions for all personnel within the operational chart ‐ Command weaknesses observed in certain exercises
	Capacity building and development of technological infrastructure	‐ Use of emerging technologies in exercise design and assessment ‐ Fixes for wireless communication issues ‐ Implementation of smart systems for damage and casualty assessment	‐ Need for intelligent risk assessment software ‐ Application of artificial intelligence for hazard design and evaluation in exercises ‐ Digitalization of operational processes ‐ Radio communication failure during the exercise
	Reforming structures and processes	‐ Analysis of exercise outcomes and subsequent redesign ‐ Revision and modification of operational plans post‐exercise ‐ Provision of feedback tailored to the exercise type	‐ Conducting exercises without feedback or corrective evaluation ‐ Implementing phased exercises with systematic output analysis ‐ Lack of effective post‐exercise documentation
	Exercise safety and security	‐ Ensuring participant safety and security ‐ Provision of Personal Protective Equipment (PPE) ‐ Identification of health hazards threatening participants	‐ Establishing a code for exercise termination in the event of a real incident ‐ Injury to personnel and damage to ambulances during the exercise ‐ Training on the proper use of personal protective equipment (PPE)
	Planning and standardization of field guidelines	‐ Formulation of exercise strategy and objectives ‐ Necessity of a preliminary plan for the exercise	‐ Development of a hazard preparedness plan ‐ Necessity of maintaining an annual exercise program ‐ Designing tabletop and operational exercises throughout the year

Empowerment and self‐efficacy enhancement of exercise personnel	Operational experience and intersectoral collaboration	‐ Engagement of experienced personnel in exercise design and execution ‐ Utilization of specialized evaluators during exercise execution ‐ Insufficiency of specialized expertise among operational forces	‐ Need for an evaluation team during exercises ‐ Lack of expert consultants in exercise design ‐ Engagement of specialized evaluators with real experience in exercises
	Improving operational functions and indicators	‐ Training and enhancement of operational functions within the exercise ‐ Reinforcement of knowledge and awareness regarding operational functions	‐ Operational training for personnel during exercises ‐ Conducting role‐based training sessions
	Innovative and creativity‐based training	‐ Conducting briefing and empowerment sessions before the exercise ‐ Utilization of educational aids and training equipment	‐ Development of a toolkit for evaluating various types of exercises ‐ Utilization of cutting‐edge technologies in exercise implementation ‐ Conducting briefing and capacity‐building sessions prior to exercises ‐ Use of simulation‐based educational tools
	Psychological support in the exercise environment	‐ Emphasis on the importance of mental health ‐ Attention to occupational burnout ‐ Encouraging voluntary participation in exercises and avoiding mandatory attendance	‐ Fostering motivation and providing psychological support for nurses ‐ Impact of excessive work shifts on optimal exercise performance ‐ Identification of capable and willing personnel for participation in exercises

Improvement and development of effective exercise drivers	Policymaking and establishing legal requirements	‐ Deficiencies in emergency policy‐making ‐ Misalignment of peripheral plans with upstream documents	‐ Absence of legal mandates for exercise design and execution ‐ Lack of legal mandate for exercise design and implementation ‐ Absence of budget allocation for exercises in financial planning ‐ Approval of a national exercise calendar
	Realism and professional ethics	‐ Necessity of realism in exercise design ‐ Utilization of risk assessment results and lessons learned in design ‐ Control of emotions in critical situations	‐ Exercises should be designed realistically rather than as mere demonstrations ‐ Mismatch between exercise conditions and real earthquake scenarios
		‐ Integration of professional ethics training into exercises	‐ Scientific and reality‐based scenario development ‐ Flexibility and adaptability of personnel to unexpected situations during exercises
	Cultural appropriateness and adaptation	‐ Lack of localization of execution guidelines to fit the national structure ‐ Inconsistency between exercise execution methods and organizational culture ‐ Discrepancy between exercises and actual infrastructure	‐ Differences in prehospital emergency organizational structure compared to other countries ‐ Mandatory use of international guidelines within a differing national framework

#### 3.2.1. Category 1: Strategic Organization of the Exercise

The key components identified within this category included the exercise command chain, capacity building and technological infrastructure development, structural and process reforms, exercise safety and security, and the planning and standardization of field guidelines.

##### 3.2.1.1. Subcategory 1‐1: Exercise Command Chain

A coherent and well‐defined operational structure is essential for an effective response to incidents and disasters, as well as for health‐focused exercises. A clearly defined command chain plays a critical role in reducing and eliminating redundant efforts.

One of the prehospital emergency managers stated: *“Effective earthquake response requires defined roles and active participation during drills, as unengaged members cause command discoordination in real events” (P2).*

An operational paramedic nurse commented: *“Exercises must practice the activation, deployment, and equipping of the Incident Command Post (ICP), which is essential for coordinating field units and the EOC during severe earthquakes” (P4).*

##### 3.2.1.2. Subcategory 1‐2: Capacity Building and Development of Technological Infrastructure

Technological interventions in prehospital earthquake exercises encompass the adoption of intelligent equipment, the digitalization of operational processes, and the reinforcement of communication networks. Furthermore, these measures involve the deployment of advanced forecasting tools, including precision damage assessment instruments, intelligent casualty estimation systems, and integrated information management platforms.

One interviewee remarked: *“To prevent operational gaps and financial inefficiencies, exercises must be supported by a well-defined logistics plan. The unrestricted deployment of all assets and the use of unqualified personnel have previously resulted in critical shortages and the wastage of resources” (P8).*

Another interviewee added: *“Integrating AI into exercise design could revolutionize disaster health management. By modeling structural and demographic variables, AI can instantaneously estimate resource gaps and create highly realistic simulation scenarios for personnel training” (P3)*.

##### 3.2.1.3. Subcategory 1‐3: Reforming Structures and Processes

The framework for earthquake response exercises comprises the design, evaluation, and implementation of improvement plans, utilizing specialized teams aligned with specific competencies. Core components include the development of reality‐based scenarios, comprehensive documentation, the application of both discussion‐based and operations‐based formats, and the recurrence of drills. Furthermore, the process involves evaluation mechanisms that generate feedback tailored to the specific exercise typology.

An operational paramedic nurse stated: *“Exercise must adhere to a standardized model with realistic, expert-driven scenarios. It is critical that all operational injects and logistical prerequisites are fully established prior to implementation” (P5).*

Another participant added: *“To ensure validity, scenarios must be developed by experienced staff and grounded in reality. This entails defining specific seismic characteristics and integrating actual data on system capacities and vulnerabilities” (P2).*

##### 3.2.1.4. Subcategory 1–4: Exercise Safety and Security

The selection of a location for an earthquake exercise is critical and must be based on the exercise objectives while ensuring the health and safety of all participants. It is imperative to obtain the necessary legal permits for the exercise site, secure the perimeter to prevent the entry of unauthorized individuals, and conduct a thorough security risk assessment to guarantee a safe training environment.

An operational paramedic nurse noted: *“Issues related to the health and safety of prehospital emergency teams are sometimes neglected. It is essential to plan for necessary interventions in case a real incident occurs during the exercise, and the safety officer must clearly define prohibited actions” (P4).*

Another participant remarked: *“Operational teams are often dispatched to the site without any prior assessment of the safety and security threats in the exercise environment” (P6)*.

##### 3.2.1.5. Subcategory 1–5: Planning and Standardization of Field Guidelines

The planning framework for earthquake exercises encompasses senior management engagement, defining priorities and expected outcomes, long‐term scheduling, and resource management. Methodological components include the utilization of hazard maps, risk priorities, and evidence‐based data from previous exercises to develop standardized field guidelines. Additionally, the process involves translating after‐action report recommendations into measurable action items within postexercise improvement plans.

One Administrative Paramedic nurse commented: *“It is crucial that the exercise plan is developed based on a comprehensive risk assessment. Currently, exercises are often planned without a regional hazard analysis or prioritization of risks, and the repetition of drills is not based on identified needs”* (P6).

Another interviewee stated: *“Although plans are updated annually, exercises frequently do not reflect these strategic objectives. Therefore, exercises should be deliberately structured to bridge the gap between documented preparedness and actual practice” (P7).*

#### 3.2.2. Category 2: Empowerment and Self‐Efficacy Enhancement of Exercise Personnel

Personnel‐focused interventions in prehospital earthquake exercises involve the enhancement of staff empowerment and self‐efficacy, the provision of continuous, multimodal training, and the implementation of incentive mechanisms. Key constituents of this domain include operational experience, intersectoral collaboration, the modification of operational functions, innovative training methodologies, and the integration of psychological support.

##### 3.2.2.1. Subcategory 2‐1: Operational Experience and Intersectoral Collaboration

The operational framework of prehospital earthquake exercises incorporates operational experience and intersectoral collaboration. Key elements include the participation of personnel with prior history in crises and drills, alongside the establishment of interorganizational coordination and multiagency interaction.

One interviewee noted: *“Establishing coordination provides a suitable foundation for effective exercises, but achieving this coordination among different organizations is challenging. Individuals with repeated exercise experience are more successful in interacting and coordinating with other agencies”* (P11).

Another participant stated: *“Joint exercises are essential for enhancing technical skills and intersectoral communication. They provide the necessary platform to identify and rectify coordination gaps between agencies before an earthquake occurs” (P10).*

##### 3.2.2.2. Subcategory 2‐2: Improving Operational Functions and Indicators

The objectives of prehospital earthquake exercises encompass the promotion of learning, personnel role practice, and organizational response capacity. The methodological framework integrates operations‐based simulations and discussion‐based formats, focusing on the enhancement of operational functions and performance indicators.

One Administrative Paramedic nurse observed: *“During an earthquake drill, we realized that our operational teams had weaknesses in ambulance staging, zone designation, triage, and patient transport to hospitals” (P7)*.

Another interviewee explained: *“High-level coordination is often compromised when participants lack thorough familiarity with operational plans. Therefore, exercises should explicitly test these frameworks to ensure staff can effectively navigate the complexities of decision-making and logistics during an actual earthquake” (P8)*.

##### 3.2.2.3. Subcategory 2‐3: Innovative and Creativity‐Based Training

Key components of practical training include the use of digital tools, the development of a standardized exercise toolkit, and conducting tailored training sessions before, during, and after the exercise that are specific to the roles of the participants.

An Administrative Paramedic nurse emphasized: *“Personnel must be thoroughly briefed on their responsibilities prior to exercises. Current training weaknesses are exposed when teams face complex tasks, such as dispatching, without adequate prior instruction*” *(P10).*

Another participant remarked: *“Training is paramount; a well-trained workforce is the key to our success in real incidents and disasters. In one prehospital earthquake response exercise I attended, the team did not know how to set up a staging area because they lacked practical, role-based training”* (P2).

##### 3.2.2.4. Subcategory 2‐4: Psychological Support in the Exercise Environment

The context of prehospital personnel is characterized by high‐stress operational environments and limited opportunities for psychological recovery. Within simulated earthquake exercises, prevailing factors include psychological pressure deriving from performance evaluation, accumulated fatigue, and mandatory participation protocols, alongside the integration of psychological support mechanisms.

One Operational paramedic nurse stated: *“Psychological resilience is as critical as technical execution. To ensure success, exercises must integrate mental preparation, real-time support for novices, and structured postdrill debriefing to mitigate stress” (P11).*

Another interviewee added: *“Mental health management is crucial. Teams require targeted training in emotional regulation for mass-casualty incidents, and psychological profiling is essential to proactively identify and support personnel at risk” (P5).*

#### 3.2.3. Category 3: Improvement and Development of Effective Exercise Drivers

The domain of exercise drivers for prehospital earthquake preparedness encompasses the establishment of legal mandates, the alignment of programs with national policy documents, and the provision of required resources. Additional components include institutional commitment, the utilization of standardized, reality‐based scenarios, and the cultural adaptation and localization of regulatory models.

##### 3.2.3.1. Subcategory 3‐1: Policymaking and Establishing Legal Requirements

The execution of effective and efficient prehospital exercises necessitates robust policymaking and the establishment of precise legal requirements. Formalizing these requirements through documented plans and programs ensures alignment with higher‐level national policies and guarantees the continuity of training activities.

An operational paramedic nurse explained: *“A national mandate is required for all prehospital services to establish a precise, annual exercise framework. Given our hazard landscape, earthquake scenarios must be the central priority of these approved response plans” (P5).*

An Administrative Paramedic nurse commented: *“Underinvestment in preparedness remains a significant weakness. The prevailing reactive mindset among officials hinders the establishment of necessary coordination frameworks prior to a disaster” (P8).*

##### 3.2.3.2. Subcategory 3‐2: Realism and Professional Ethics

The design framework for prehospital earthquake exercises incorporates the utilization of previous experiences, lessons learned, and after‐action reports. Key elements include the formulation of scenarios reflecting operational realities and environmental conditions and the application of risk analysis for problem‐focused exercise design. Furthermore, the domain involves the integration of professional ethics, comprising the preservation of casualty dignity, emotional management, and care provision under constraints of pressure and limited resources.

One Administrative Paramedic nurse stated: *“It is crucial that an earthquake exercise is not just for show but is based on addressing real deficiencies. Every exercise must have a scenario, injects, and a background story”* (P1).

An operational paramedic nurse remarked: *“We do not need to incur high costs to improve preparedness, which can lead to conducting ceremonial drills that are only useful for documentation but do not actually improve readiness”* (P4).

Another participant added: *“There is a clear disconnect between our training content and the needs of disaster exercises. The exercise scenario must be based on reality, and the drill should be conducted in earthquake-prone areas”* (P2).

##### 3.2.3.3. Subcategory 3‐3: Cultural Appropriateness and Adaptation

The contextualization of prehospital emergency exercises encompasses the adaptation of regulations, guidelines, and structures to specific cultural, legal, and infrastructural frameworks, alongside the alignment of international guidelines with organizational and societal cultural norms.

An operational paramedic nurse observed: *“The failure to adapt international standards to the local context results in purely performative drills, which in turn lead to disengagement and diminished motivation among personnel*” *(P11).*

Another interviewee said: *“We cannot simply copy international protocols; our capabilities and public culture differ fundamentally. Attempting to apply these models without localization causes*.*”*

## 4. Discussion

This study aimed to identify the key components for the design, implementation, and evaluation of prehospital exercises for earthquake hazards. The analysis revealed three primary categories of indicators: “Strategic Organization of the Exercise,” “Empowerment and Self‐Efficacy Enhancement of Exercise Personnel,” and “Improvement and Development of Effective Exercise Drivers.”

### 4.1. Category 1: Strategic Organization of the Exercise

The strategic organization of exercises is fundamental to enhancing team cohesion and rapid response capabilities. According to participants, one of the important standards and indicators of the exercise is the Exercise Command Chain. The study’s findings emphasize the importance of having a clear hierarchical structure and defined roles. This clarity is vital for effective coordination, reducing duplication of efforts, and enabling quick decision‐making during prehospital earthquake responses. Such an organization ultimately improves readiness and resilience in disaster health management systems. The main points reiterate that a well‐structured command chain is crucial for enhancing operational efficiency in emergencies, particularly during earthquakes. Different research about this finding shows that the Incident Command System (ICS) is crucial for emergency response training, as it improves communication, clarifies authority, and enhances task management, thereby improving team cohesion and decision‐making in disasters [21, 22]. Capacity building and development of technological infrastructure are important issues that have received less attention in the exercises. From the participants’ point of view, the integration of intelligent disaster decision‐support systems (IDDSS) has emerged as a critical component of modern preparedness planning. The integration of IDDSS has emerged as a critical component of modern preparedness planning. Advances in artificial intelligence have significantly enhanced decision‐making and risk assessment. Natasha Sanchez Cristal emphasized the necessity of integrating simulation‐based exercises into health emergency management training programs [23]. Different studies also showed that predictive analytics and geospatial modeling help emergency planners create better hazard‐specific scenarios and improve assessments. High‐fidelity simulation tools enhance earthquake training by offering realistic experiences. These simulations boost performance, teamwork, and decision‐making skills during emergencies [24, 25].

The authors of the present study contend that the effectiveness of an earthquake exercise is significantly enhanced through meticulous planning, clear objectives, the use of realistic scenarios, and the integration of modern technologies such as artificial intelligence. Simulation‐supported exercises significantly raise performance quality by fostering teamwork, improving risk recognition, and reducing cognitive load during decision‐making. Designing earthquake exercises with a strategic and systemic perspective will lead to increased operational readiness, a reduction in human error, and enhanced resilience against disasters.

### 4.2. Category 2: Empowerment and Self‐Efficacy Enhancement of Exercise Personnel

Empowerment and self‐efficacy enhancement of exercise personnel was another main category that was extracted in this study. Empowerment and self‐efficacy impact prehospital nursing preparedness. Ongoing training and incentives improve performance, while role confusion and inadequate training hinder readiness. The findings revealed that sustained opportunities for learning, combined with structured operational drills and psychological support, direct field experience in disaster response, and significantly improved operational performance and decision‐making capacities. Paramedic nurses demonstrated substantial skill improvement through direct participation in practical sessions. These findings were consistent with the results of several studies [26, 27]. Consequently, a blended approach that combines theoretical instruction, practical application, and the use of modern technologies tailored to learning objectives is strongly advocated for empowering personnel.

One of the most important findings of this study, which was overlooked in other studies, was psychological support in the exercise environment. Addressing the mental health of operational teams, who provide services under demanding and high‐stress conditions, is a priority for any health system. Managers must establish appropriate mechanisms to mitigate stressors, such as reducing prolonged commutes, improving accommodation and rest facilities, and addressing basic needs [28]. Furthermore, psychological support from peers and strong interpersonal connections among team members can improve the quality of service delivery. A focus on mental health is an indispensable component of emergency planning and response and must be integrated into exercise design [29].

The authors of this study contend that empowering prehospital emergency personnel through innovative training methods, coupled with robust psychological support including mentorship for less experienced individuals and proactive mental health management, improves their self‐confidence and enhances their self‐efficacy in high‐stress disaster situations.

### 4.3. Category 3: Improvement and Development of Effective Exercise Drivers

The improvement and development of effective drivers is another pivotal component in the design, implementation, and evaluation of prehospital exercises. The study findings showed that gaps within governing policies and regulatory frameworks can heighten systemic vulnerability during earthquakes, underscoring the need for more coherent and future‐oriented preparedness strategies within the Iranian prehospital system. A critical aspect of this is the reallocation of resources, which must align with a policy shift from a response‐centric model to one that prioritizes prevention. This emphasizes the need for coordinated policies and resource allocation to transition from reactive to proactive paradigms [30]. Evidence from recent studies, including the work of Nikjoo et al., demonstrated that investment in emergency management programs can significantly elevate preparedness levels [31]. Another noteworthy consideration for enhancing response quality is the integration of ethical principles into disaster training and the development of practical guides for existing protocols. Ethical tenets such as respecting the dignity of the injured, managing emotional responses, and providing appropriate care under conditions of extreme psychological pressure and resource scarcity must be explicitly articulated, and actionable guidelines must be available for implementation [32, 33]. It is also critical to recognize that many international prehospital emergency guidelines, developed in high‐income countries, are often not suitable for the cultural contexts, resource limitations, and training levels of developing nations with limited resources [34, 35].

The authors argue for using past earthquake experiences to improve current practices. They suggest holding ethics workshops on human dignity and creating a practical Ethics Field Manual as tools for local application. Policymakers should reform laws and policies to eliminate barriers to annual exercises. Differences in emergency service structures and legal frameworks create challenges that require legal reforms aligned with disaster risk reduction strategies.

### 4.4. Limitations and Strengths of the Research

A primary strength of this study is its use of qualitative content analysis to identify the essential components and indicators for conducting practical prehospital exercises. The identification of these factors is crucial for enhancing the quality of such drills. Another key strength was the purposive sampling strategy, which ensured that interviews were conducted with individuals who possessed direct, hands‐on experience in implementing exercises.

The study is not without limitations. First, the collected data were reliant on the experiences and recall of the interviewees, introducing the potential for recall bias. To mitigate the risk of distortion or loss of detail, efforts were made to contact and interview participants promptly after receiving ethical approval. Second, although strategies such as member checking, peer debriefing, and team consensus were employed to enhance credibility, the potential for interviewer bias and subjective interpretation remains. Third, this study offers valuable insights into the in‐depth experiences of Paramedic Nurses from the Emergency Medical Services within the EMS system. However, it must be acknowledged that the generalizability of these findings to other international settings is limited. The design, implementation, and evaluation of disaster management exercises, whether discussion‐based or operations‐based, are heavily contingent upon the training infrastructure, available resources, and workforce capacity of specific health systems. Consequently, conducting similar research across diverse geographical and sociodemographic contexts is recommended to yield a more comprehensive understanding of Paramedic Nurses from the Emergency Medical Services regarding disaster exercise management. Finally, as the study was conducted at a national level, broader international comparisons or the generalization of policy recommendations should be approached with caution.

### 4.5. Practice and Policy Implications

The findings of this study are organized into three main themes, each with direct implications for practice and policy. The first theme, strategic organization of the exercise, underscores the importance of training in and strengthening the incident command structure. It proposes the development of innovative educational methods as a solution to overcome the challenges associated with exercise implementation. The second theme, empowerment and self‐efficacy enhancement of exercise personnel, emphasizes the need for standardized training and exercise design. It also brings to the forefront the critical, yet often neglected, importance of addressing the psychological skills and well‐being of responders. The third theme, focused on the localization of exercises, highlights the necessity of understanding organizational differences and adapting international guidelines to the local context to ensure their relevance and effectiveness.

## 5. Conclusion

This research establishes a crucial knowledge framework that empowers exercise planners in prehospital settings to design scientifically sound and standardized exercises aimed at enhancing disaster response processes. The primary finding is that the successful implementation and evaluation of both discussion‐based and operation‐based exercises, when informed by these identified quality indicators, significantly fosters knowledge development and promotes essential behavioral change among prehospital paramedic nurses, thereby facilitating a standardized emergency response.

A key actionable implication is the necessity to embed the enhancement of psychological abilities, including stress management and perceived self‐efficacy, directly into program design. Furthermore, while the integration of modern technologies unequivocally enhances exercise quality, sustained obstacles persist, notably the shortage of sustainable logistical/financial resources and skilled human capital for continuous implementation. Therefore, prehospital systems must proactively establish mechanisms for securing sustained financial resources and ensuring the continuous evaluation of capable personnel. Future research is strongly recommended to quantitatively assess the long‐term effectiveness of these technology‐enhanced training programs, specifically evaluating the impact of digital scenario simulation and interprofessional education on teamwork and the standardization of postexercise evaluations to rigorously benchmark performance gaps and cost‐effectiveness. Ultimately, prioritizing the integration of nontechnical cognitive skills such as stress reduction and improved decision‐making remains paramount for maintaining functional readiness under resource constraints.

## Author Contributions

A.A.: conceived the study, prepared the analysis plan, conducted the analysis, and prepared the draft manuscript. H.F.: conceived the study, prepared the analysis plan, performed the literature search, screened for study inclusion/exclusion and risk of bias assessment, conducted the analysis, and prepared the draft manuscript. M.N., M.A., and A.K.: prepared the analysis plan, performed the literature search, screened for study inclusion/exclusion, assessed the risk of bias, and reviewed the manuscript. A.A. and H.F.: prepared the analysis plan and reviewed the manuscript. A.T. and A.S.: prepared the analysis plan and reviewed the manuscript.

## Funding

This research received no specific grant from any funding agency in the public, commercial, or not‐for‐profit sectors.

## Disclosure

All authors approved the final manuscript.

## Ethics Statement

The Ethics Committee of Kerman University of Medical Sciences approved this study. The ethical approval code is IR.KMU.REC.1403.522. All methods were performed in accordance with the relevant guidelines and regulations; this article does not contain any studies with animals performed by any of the authors. Informed consent was obtained from all individual participants included in the study, and written informed consent was obtained from individual participants. Confidentiality and anonymity of the participants were ensured by coding the questionnaires. Study participants were clearly informed of their freedom to opt out of the study at any time without justification.

## Conflicts of Interest

The authors declare no conflicts of interest.

## Data Availability

The data that support the findings of this study are available from the corresponding author upon reasonable request.
